# Long-Term Adverse Effects After Electroconvulsive Therapy (ECT): A Narrative Analysis Exploring People’s Experiences, Meaning-Making, and Coping

**DOI:** 10.1177/10497323241303391

**Published:** 2024-12-09

**Authors:** Emily Shipwright, David Murphy

**Affiliations:** 1 School of Psychology, Faculty of Health, 6633 University of Plymouth, Plymouth, UK

**Keywords:** electroconvulsive therapy, ECT, adverse experiences, long-term experiences, lived experience, narrative analysis, iatrogenic harm, memory loss, ambiguous loss

## Abstract

Approximately 2500 people receive electroconvulsive therapy (ECT) annually in the United Kingdom; however, there is growing evidence of long-term adverse effects. Few studies have focused on people’s experience of ECT, particularly over the long term. The experience of ECT is known to be complex, requiring qualitative exploration. Therefore, this study aimed to explore the long-term adverse experiences of ECT, including psychological impacts, meaning-making, and coping. Narrative analysis considered thematic, structural, and performative elements of seven people’s stories. Themes were explored across the timeline of participants’ experiences. Before ECT, participants felt misinformed regarding ECT and misunderstood by healthcare professionals. They noticed immediate changes in their cognition, memory, and mood after ECT. Returning home was important in participants’ discovery of differences. Long-term impacts were loss (of ability, memory, humanity, and connection), the realization that ECT had been damaging, and understanding ECT to have caused brain damage. The extensive nature of loss experienced by participants was comparable to the concept of ambiguous loss. They experienced a lack of follow-up care and denial of their experiences, which could contribute to psychological iatrogenic harm. Participants coped with adverse experiences by using prompts and strategies and connecting with others. Further research is needed into the adverse long-term effects of ECT, especially considering cognitive effects, memory loss, and how these contribute to a changed sense of self. Service development is urgently required, especially for ECT follow-up care.

## Introduction

Electroconvulsive therapy (ECT), the administration of an electric current to induce seizure activity in the brain, has been used as a mental health treatment since 1938 (Bini & Cerletti, 1938/2013). The practice, utilization, and administration of ECT varies globally (Leiknes et al., 2012), with an estimated 2500 people receiving ECT annually in the United Kingdom (Read et al., 2021). Despite ECT’s longevity of use and decades of research, the relative contributions of numerous proposed mechanisms of action remain unclear (Singh & Kar, 2017). Furthermore, the ethical use and long-term effectiveness of ECT are debated (González-Pando et al., 2021).

Within the United Kingdom, ECT is a recommended treatment for severe depression, severe mania, or catatonia only when other treatment options are ineffective or the condition is life threatening (National Institute for Health and Care Excellence [NICE], 2009). However, there are ongoing concerns about the standards of care and monitoring of ECT in the United Kingdom (NICE, 2009). In the United Kingdom, information leaflets provided to patients regarding ECT have been found to minimize the prevalence or severity of memory loss and largely omit the limited nature of ECT’s long-term benefits (Harrop et al., 2021; Read et al., 2023). Raising concerns about the extent to which patients consent for ECT is truly informed.

### Long-Term Adverse Effects

Following ECT, there is a possibility of permanent adverse effects including memory and cognitive impairment (Robertson & Pryor, 2006). Very little is known about the neurobiological underpinnings of these effects (Nobler & Sackeim, 2008). Furthermore, the Royal College of Psychiatry has described the routine clinical tests used to monitor the cognitive effects of ECT as neither valid nor reliable nor repeatable (Waite & Eaton, 2013). González-Pando et al. (2021) suggest that research into the long-term effects of ECT is lacking and that adverse effects imply a profound disturbance of lived experiences. Iatrogenic harm, the infliction of harm through medical interventions, is a known possible outcome of ECT (Dubey, 2017). Hailey et al. (2015) conducted a questionnaire of ECT patients in England and Wales, and almost 20% of respondents reported experiencing severe and enduring memory loss. In their audit of ECT in England, Read et al. (2021) were unable to obtain any National Health Service (NHS) data on efficacy or adverse effects beyond the end of ECT treatment, despite the known possibility of harm. The long-term adverse effects of ECT lack monitoring and are under-researched.

### Lived Experience

There is a small amount of qualitative research exploring people’s accounts of their ECT experience. This research generally focuses on the short time periods leading up to, during, or following ECT treatments (Ejaredar & Hagen, 2014). Even fewer studies explore long-term impacts (Wells et al., 2021). People’s accounts of ECT are mixed, with different factors, such as feeling comfortable and trusting in staff, contributing to more positive accounts, whereas more negative accounts include experiencing ECT as frightening and illogical (Knight et al., 2017). These studies have emphasized the need for further inquiry (Coman & Bondevik, 2023; Rose et al., 2004), which utilizes qualitative research methods, thus enabling exploration of the complexity of experience (Rose et al., 2003).

Wells et al. (2022) used qualitative methodology to propose a framework that outlines the key factors involved in how people experience ECT and its long-term impacts. They found that people’s experience of ECT varied greatly and was influenced by key factors such as decision-making experiences and post-ECT clinical support. Furthermore, the long-term impacts of ECT could be split into its direct impacts on a person’s cognition and emotional experience, and how these impacts affect people’s daily activities, relationships, and ongoing healthcare. Finally, they found that people described developing multiple strategies to cope with the long-term negative impacts that ECT had on their lives. Wells et al. (2022) add to previous research describing the emotional impacts of ECT (Johnstone, 1999; Koopowitz et al., 2003; van Daalen-Smith, 2011). Although providing an important theoretical framework mapping the long-term experiences of ECT, Wells et al. (2022) were limited in their exploration of the psychological effects, including meaning-making.

The adverse psychological experience of ECT has been the primary focus of just one empirical study, which found that ECT can carry deeply symbolic meanings (Johnstone, 1999). Furthermore, Koopowitz et al. (2003) concluded that to address the psychological consequences of ECT, further research needs to explore the perception of memory loss and how this impacts the sense of self following ECT. Therefore, to build upon the work of Wells et al. (2022), there is a need for research utilizing qualitative methodology that explores the long-term impacts of adverse effects of ECT to include an in-depth exploration of psychological effects and meaning-making.

## Aims

This study aimed to explore the stories of people who report experiencing long-term adverse effects from ECT, including psychological impacts, meaning-making, and coping.

## Methods

### Narrative Research

This study utilized a narrative approach to explore how people organize and make sense of their experiences and memories through storytelling (Bruner, 1991). Narratives can be seen as a human strategy for processing and coming to terms with change over time (Herman, 2007). The use of narrative methodologies can highlight how individuals view themselves and the world and how they make meanings from their experiences (Labov & Waletzky, 1967). Other aspects of storytelling, such as story structure and performance, including coherence, are also examined through narrative analysis (McAdams & McLean, 2013). Narrative coherence refers to the extent to which a story makes sense to the listener and is thus able to convey the content and meaning of the described events (Vanaken et al., 2021). Therefore, narrative analysis is a salient methodology for exploring the complexity of long-term adverse experiences of ECT.

### Researcher Position

Narrative analysis acknowledges that researchers cannot directly access participants’ experiences; therefore, every stage of the research process requires representational decisions (Riessman, 1993). In acknowledging the level of interpretation and influence of the researcher, it is important to consider my position here. (Author ES) My interest in ECT has been influenced by my role as an NHS trainee clinical psychologist and a prior job role working with people who have experienced ECT. I hold a critical view of the biomedical assumptions underpinning ECT; I also recognize that some people report it to be beneficial. To clarify my thoughts and assumptions about ECT and to monitor how my position impacted my interpretation, I completed a presuppositional interview (Barrett-Roger et al., 2023) and maintained a reflective journal (Ortlipp, 2008). I also used supervision and peer supervision to enhance reflexivity.

### Consultation

To ensure that the research was both meaningful and sensitive to participants, two people with lived experience of long-term effects of ECT were consulted. Consultees contributed to the development of the recruitment strategy, the use of timelines, research prompts, and the structure of a follow-up interview.

### Recruitment

Participants were recruited through independent ECT researchers who distributed the research flyer to online mental health advocacy groups and websites, and an online ECT support group. Ten people expressed an interest in participating, one person chose to withdraw prior to giving consent, and two people were excluded due to not receiving ECT in the United Kingdom. Therefore, seven people were recruited. Following common narrative analysis practices (Frost, 2021) and to be comparable to other narrative studies of ECT experiences (Ejaredar & Hagen, 2014), the sample size was kept small to allow for in-depth analysis of rich data.

### Inclusion Criteria

Adults (aged over 18 years) who considered themselves to be experiencing at least one adverse effect of ECT were recruited. Participants had to have received ECT treatment in the United Kingdom, not to be currently undergoing ECT, and to have received their last ECT treatment a minimum of 6 months and a maximum of 20 years prior to the first research interview. Participants confirmed that they were not currently experiencing a mental health crisis and that they felt able to cope with any distress that may arise from discussing their experiences. Not meeting these criteria resulted in exclusion from the study.

### Participants

Table 1 presents basic information about the sample characteristics. All participants identified as White: English, Welsh, Scottish, Northern Irish, or British. All participants received bilateral ECT and reported that they had a diagnosis of depression; for some, this was part of their primary diagnosis (five), whilst for others this was included within a list of diagnoses that they had received (two). One participant received ECT involuntarily whilst detained under the Mental Health (Care and Treatment) Act (Scotland) (2003). Another participant received some ECT whilst detained under the Mental Health Act (1983). Due to vague medical notes and an absence of memory, this participant is not sure whether they consented to all ECT sessions. The other five received ECT as voluntary patients.

**Table 1.** Demographic Characteristics and Treatment History of Participants.

	*n*	%
Gender		
Female	6	86
Male	1	14
Non-binary	0	0
Age (years)		
30–39	1	14
40–49	4	57
50–59	1	14
60–69	1	14
Time since last ECT (years)		
0–5	2	29
6–10	1	14
11–15	2	29
16–20	2	29
Total number of ECT sessions received		
0–19	4	57
20–39	0	0
40–59	1	14
60–79	1	14
80–99	0	0
100–119	0	0
120–139	0	0
140–159	0	0
160–179	0	0
180–199	1	14

### Ethics

This study received full ethical approval from the University of Plymouth Faculty of Health Research Ethics and Integrity Committee (Project ID 3939). Participants responded to an advert containing details about the research and were subsequently sent additional information about the study. Prior to enrollment in the study, written consent was obtained by the lead researcher. Confidentiality, anonymity, right to withdraw, and possible risks of participating were outlined before the narrative interview. Participants were offered a £15 voucher as a recompense for their time; not all accepted this and instead asked that it be donated to charity. Debrief information containing researcher details, a reminder of the research aims, and details for support organizations were distributed prior to the narrative interview commencing. Follow-up interviews also offered some space for debriefing. To ensure anonymity, all names used in this study are pseudonyms.

### Procedure

Data was collected across two interviews: a narrative interview (Reissman, 2008) and a follow-up interview, which took place approximately a month apart. The narrative interview was the primary data collection method. Each interview began by restating the research aims and creating a basic timeline of the participant’s experience with ECT. The following narrative question was then asked: “I would like you to tell me about your experiences of ECT and following. Please take as much time as you need.” Following this question, the researcher used prompts to explore, expand upon, and understand participants’ stories.

Although optional, every participant took part in the follow-up interview. These interviews started with a summary of the key story themes from the participant’s first interview. Participants were invited to elaborate on, clarify, or correct any themes described and to add additional information or reflections. The changes to narratives following second interviews were limited, with only minor amendments or clarifications being made. Although this process will enhance analysis, narratives will continue to be impacted by researcher bias.

Five interviews took place on a video calling platform and two took place via telephone according to participant preference. All were audio recorded. Narrative interviews lasted between 54 and 102 minutes, and follow-up interviews were 22–84 minutes long.

### Transcription and Analysis

Interviews were transcribed verbatim by the lead researcher; names were changed, and identifiable details were removed to ensure anonymity. Transcripts were subject to repeated readings to enable familiarization with the data. Sections of transcripts were analyzed jointly during individual supervision and group peer supervision to strengthen the validity and increase rigor of analysis (Birt et al., 2016). The use of a second interview served in part as member checking, enhancing the rigor of findings.

Narrative analysis followed Riessman’s (2008) framework utilizing three narrative approaches: thematic analysis (what was said), structural analysis (how the story was told), and performative analysis (how the talk was interactively produced and performed) to strengthen and triangulate analysis.

#### Analysis of Story Themes

Each transcript was individually reviewed, and a timeline of each participant’s experience was added to. Both common and unique aspects of experiences were searched for. Narrative interviews were considered the primary data for analysis from which key story themes were identified. Key story themes were compared and synthesized over data sets to create overarching themes; this process was assisted using NVivo 12 software (QSR International, 2017).

#### Structural and Performative Analysis

Following the analysis of story themes, each transcript was analyzed considering structural and performative elements (Reisman, 2008). Structural narrative analysis was informed by Labov and Waletzky’s (1967) and Baerger and McAdams’ (1999) frameworks. These different frameworks guided the exploration of narratives by considering and exploring the presence or absence of different structural elements, for example, orienting clauses or clauses integrating meaning. For performative aspects, stories were considered in terms of the context in which they were told. This included consideration of what participants were trying to communicate, how they were conveying this information, and how this may influence the story told (Labov, 1982; Reisman, 2008).

## Results

Results are presented in three parts. Part 1 contains a brief summary of each participant’s story of ECT. Part 2 presents key story themes across all participants’ narratives, depicting shared experiences and highlighting key differences. Part 3 explores structural and performative elements of how participants told their stories.

### Part 1: Individual Stories of ECT

#### Charlotte

##### Six Courses, 74 Sessions of Bilateral ECT. First–Last Treatment: Oct 2014–March 2020

Charlotte was desperate to help her depression, so she consented to ECT. She experienced an improvement in mood from her first and second ECT courses but little to no improvement in subsequent courses. During and following the final two ECT courses, she became aware of significant autobiographical memory loss and difficulties retaining information, later confirmed by neuropsychological tests. A key part of Charlotte’s identity has been her intellect; however, she can no longer work as an academic researcher. Since ECT, she continues to discover autobiographical memory loss.

#### Dean

##### One Course, Four Sessions of Bilateral ECT. First–Last Treatment: Dec 2021–Jan 2022

Dean experienced low mood following a cancer diagnosis. Whilst detained in a psychiatric hospital under the Mental Health Act, Dean was told one morning not to eat breakfast because he would be having ECT. Dean did not consent. However, despite his distress, he received four sessions of ECT. Following discharge, Dean heard a radio program about ECT, which brought back traumatic memories and anger about his treatment. To help him process his experiences and raise awareness, Dean has published about his ECT.

#### Flora

##### 12–14 Courses, 189 Sessions of Bilateral ECT. First–Last Treatment: March 2000–March 2008

Flora was admitted to a psychiatric hospital, experiencing severe burnout, which was diagnosed as depression. She was assured that ECT was not dangerous and that memory loss would be temporary. Flora consented, fearing repercussions if she refused. Following her first ECT course, Flora developed an eating disorder. Cycles of ECT followed where she would return to a safe weight, be discharged, relapse, and return to hospital for further ECT. Flora is unsure of exactly how many courses of ECT she has received. During ECT, Flora became aware of autobiographical memory loss, and on returning home, significant short-term memory loss became apparent; both have persisted.

#### Harriet

##### One Course, Six Sessions of Bilateral ECT. First–Last Treatment: Oct 2006–Nov 2006

Harriet experienced depression and anxiety following workplace bullying. During a voluntary psychiatric hospital admission, she consented to and received ECT, which she was told might lead to mild and temporary memory loss. On returning home, short-term memory loss became apparent, the extent of which was confirmed by neuropsychological testing. Harriet described herself before ECT as a reading and learning machine; she is now unable to read or work. Following ECT, Harriet had children, and she cannot remember them growing up.

#### Paula

##### One Course, Six Sessions of Bilateral ECT. First–Last Treatment: March 2012–April 2012

Paula experienced a complex traumatic childhood. As an adult, she experienced low mood and sought help from a psychiatrist who erratically changed the psychiatric medications and dosages she was taking. As a voluntary patient in a psychiatric hospital, Paula consented to ECT. However, she did not feel she had a choice. Immediately following ECT, Paula had an increase in intrusive traumatic memories. Following discharge, she became more aware of significant short-term and significant autobiographical memory loss. Paula is unsure how much of her memory loss can be attributed to ECT alone, alongside years of treatment with psychiatric medication. Paula described herself before ECT as a voracious reader, and she is no longer able to retain enough information to read.

#### Rose

##### Five Courses, 44 Sessions of Bilateral ECT. First–Last Treatment: Jan 2012–Jan 2013

Rose experienced childhood adversity. In her late teens and twenties, she described studying and travelling to build her life. Following increased stress, Rose was admitted to a psychiatric hospital. Rose believes that she received some ECT whilst detained under the Mental Health Act and some as a voluntary patient. She cannot remember any of her ECT, relying on medical notes and friends’ accounts of this time. Rose reports experiencing significant autobiographical memory loss, including the loss of self-defining memories from travelling. Rose continues to be supported by mental health services and, despite trying, has been unable to access neuropsychological testing to help understand the impacts of ECT.

#### Stella

##### One Course, Six Sessions of Bilateral ECT. First–Last Treatment: Aug 2016–Aug 2016

Stella struggled with her mental health in her teenage years. Following this, she experienced periods of depression and suicidal thoughts. Subsequently, she was admitted to a psychiatric hospital, where she attended therapeutic groups and was prescribed psychiatric medication. Some professionals did not think these interventions were having much effect and therefore suggested ECT. Whilst in hospital, Stella noticed some short-term memory loss between ECT treatments. Stella did not realize the extent of her memory loss until she returned home, when she noticed that she was struggling to recognize people known to her. Stella now has autobiographical memory loss and difficulties with her short-term memory, which has affected her ability to cook and shop for herself.

### Part 2: Shared Themes Across Participants’ Accounts

**Figure 1.**
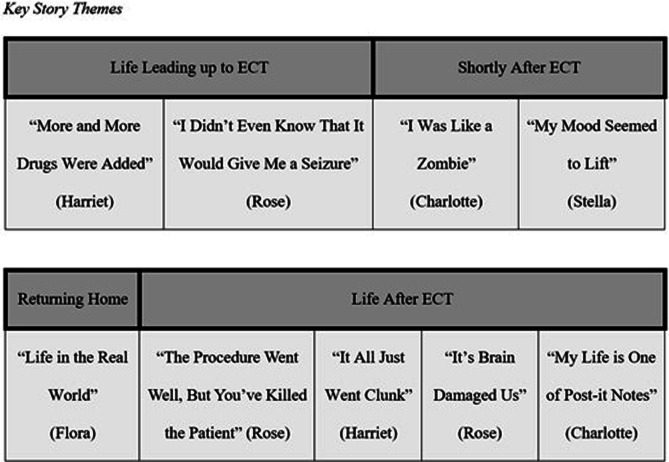
Key Story Themes.

Figure 1 outlines key story themes organized chronologically across common stages of participants’ accounts of ECT. In total, there are nine key story themes. The themes “The Procedure Went Well but You’ve Killed the Patient” and “My Life Is One of Post-It Notes” each contains three sub-themes, enabling unique aspects to be explored.

#### Life Leading up to ECT

##### “More and More Drugs Were Added” (Harriet)

Although all participants identified different contributory factors to their distress, in each story, either they or family members sought help from mental health services. Participants were given diagnoses and prescribed psychiatric medication, subsequently receiving ECT.

All of this started with workplace bullying. […] I got, you know, quite sad about all of that, went to my GP she put me on the Citalopram, the Citalopram sent me on the rollercoaster, the rollercoaster took me to ECT. (Harriet)

Furthermore, the majority of participants identified psychiatric drugs and their side effects or withdrawal as factors that worsened their presentation and the ability of psychiatric staff to understand them: “The GP prescribing Citalopram, without any recognition that it could have quite negative side effects, and so, I think those side effects were misdiagnosed and more and more drugs were added” (Harriet).

##### “I Didn’t Even Know That It Would Give Me a Seizure” (Rose)

All participants consented to all ECT treatments, except for Dean and Rose. All consenting participants, including Rose, felt that the process was flawed and that they did not understand the ECT procedure or its potential for long-term and life-changing impacts. Rose and Paula did not know that they had consented to having an induced seizure.

Paula and Flora felt pressured to consent to ECT due to the powerful position of professionals: “You know they’re kind of pushing for that treatment. You don’t feel like you can say wait or no” (Paula). On the other hand, Charlotte described her desperation to receive any treatment that would help: “When it came to trying ECT, I was utterly desperate.” Despite this initial position, when considering her experiences of permanent memory loss, Charlotte does not feel that her consent was informed: “I honestly don’t believe […] the patient information that I was given constitutes sufficient information to make an informed choice.” This was true for many participants when considering the devastating impacts that cognitive changes and memory loss from ECT had on their lives.

Dean’s experience was different to other participants. All decisions for Dean to have ECT were made without his knowledge, and he did not consent. Dean talked about his shock at learning he was to have ECT: “I was told not to go for breakfast because I would be getting ECT and that came like a bolt from the blue,” and reflected on his experiences with disbelief, incredulity, and distress: “How could they be so inhumane to do that to a cancer patient!”

#### Shortly After ECT

##### “I Was Like a Zombie” (Charlotte)

Participants’ ability to remember their experiences following ECT varied, but all struggled to recall the time immediately surrounding treatment. All participants described coming round from ECT, feeling confused and disoriented: “I woke up I didn’t know who I was, I didn’t know where I was, I didn’t know what the heck had happened” (Flora). Transient headaches and muscle stiffness immediately after ECT were frequently described by participants.

Most participants reported memory loss whilst they were receiving ECT: “The memory loss was absolutely profound. My family, they were really shocked” (Harriet). For some, this memory loss felt different to the persistent memory loss they would go on to experience: “There is this sort of mist, haze in your mind when you’re having ECT that affects your memory. […] I was like a zombie” (Charlotte).

##### “My Mood Seemed to Lift” (Stella)

Most participants generally reported a change in mood immediately following or between ECT sessions; however, this change was not always perceived as positive or lasting. Two participants described significant changes in their psychological state whilst receiving ECT, becoming aggressive or angry: “During ECT I got very aggressive […] I was never like that when I was not getting ECT” (Flora). Four participants described an initial and short-lived improvement or changed mood: “So you know, I got a glimpse of blue skies and then it just, you know, the storm clouds gathered, and I was back where I was” (Charlotte). For Paula, it was hard to say whether the change in mood was a positive experience because “with raised mood comes anxieties, past traumas,” which were intrusive and distressing, and therefore, the shift did not feel like an improvement in psychological well-being.

#### Returning Home

##### “Life in the Real World” (Flora)

For all participants, returning home from hospital following ECT and trying to re-establish their lives confronted them with the realization that cognitive abilities were impacted, short-term memory had significantly reduced in capacity, and autobiographical memories had disappeared. Short-term memory difficulties often became apparent due to changes in functional abilities.

The shorter-term stuff came more after I got out because, of course, if you’re getting constant ECT over eight years, you’re not really living a normal adult life in the real world, you know, you don’t need to use your brain in that way, but when I started you know cooking, I couldn’t follow a recipe, I forget about the pan on the stove. (Flora)

For some, returning to reading and studying made difficulties apparent: “I’d pick up a book expecting to read it and I could physically sound out the words, but I couldn’t hold a sentence, […] the shelf where you put the sentences on, where you assemble them wasn’t there anymore” (Harriet).

Furthermore, it took participants time to realize that difficulties were present and persistent.

A lot of the effects of it have come to light a lot longer, they are more clear and they weren’t obvious at the time, like it's as the time has gone on, and also, I’ve noticed that it seems to be like they’re going to be long term. It’s not, it’s not improved. (Stella)

The loss of autobiographical memories was often realized when participants encountered situations that should prompt memories or familiarity: “I didn’t realize I was engaged […] until I found some photographs” (Rose).

Due to the suggestion in the consent process that changes would be temporary, some participants waited for their abilities to return. This led to participants blaming themselves or struggling to understand the persistence of their difficulties: “I expected everything to improve and get better because I’d always been told that whatever side effects there were, would be short-term […] feeling like I’d failed because things weren’t improving” (Paula).

#### Life After ECT

##### “The Procedure Went Well, but You’ve Killed the Patient” (Rose)

In all accounts, loss from the effects or impacts of ECT was significant. This was apparent in several distinct ways captured across the sub-themes: Loss of Ability, Loss of Self, and Loss of Humanity and Connection. Overall, the experience of loss was profound and had a huge emotional impact on all participants, as described by Stella: “The experience of ECT has broken part of my heart and robbed a large chunk of my mental functioning and ability to form and enjoy memories, the very thing that makes us human.”

###### Loss of Ability

The experience of cognitive changes and memory loss had profound and far-reaching impacts: “Struggles that I have with like executive functioning and planning and trying to multi-task and recall and orientation, like I get confused in places like I can’t remember” (Stella). When short-term memory loss was experienced, it was immensely frustrating: “It’s just this intense frustration that no matter how hard I paddle, you know, I am absolutely working at, you know, my hardest to remember things” (Charlotte). The loss of autobiographical memories was particularly devastating: “Like people dying, I’ve lost my nan and grandad and my dad, and I can’t remember maybe hundreds of memories with them, that’s one of the hardest things” (Rose). Photographs that should have served as memory prompts instead caused intense distress and confusion as memories could not be recalled or even slightly recollected: “I deleted all the photos I had of the children, as you know, when they were little because […] I never get to remember any of it” (Harriet).

Discovering memory loss became a never-ending process for Stella, Harriet, and Rose due to either repeatedly encountering things that should, but did not, prompt memories or being unable to retain or recall that they had forgotten significant memories. This loss was continuous, confusing, and disorientating: “I don’t know what is going on, it’s like the world is just chaos. […] it’s almost new every day because it’s like I’m discovering it over and over” (Harriet).

###### Loss of Self

For some participants, autobiographical memory loss was so extensive that key experiences and stories that had contributed to their identity were unable to be recollected.

You know the memories that create our reality that create our framework, you know, if you stop remembering the connections you have with people and places and things that you did, where is your sense of self, where is your place? (Paula)

All participants reported that the adverse effects of ECT had fundamentally changed their ability to be themselves. Memory loss impeded participants’ ability to carry out cherished activities: “It has destroyed who I was because what else was I except that that person who read and learnt all the time” (Harriet). For Dean, the traumatic experience of having ECT changed his worldview: “Being compulsorily detained in hospital and given such a sort of a brutal treatment without my consent does shake up my sort of reality a bit, you know the world is not such a benign place after all.”

The majority of participants lost important identities that their intellect or work had previously given them: “I resigned my post; I lost that identity. I felt like I had lost part of myself because I had lost something that I prized most dearly” (Charlotte). For some participants, this change meant that they lost their envisaged future or direction: “I always felt like I had time ahead of me […] to create a career, to have something meaningful like that and now I find that I don’t have that capacity” (Paula).

###### Loss of Humanity and Connection

Participants felt that after ECT, they no longer perceived themselves as normal: “I feel like a freak, ashamed” (Flora). Additionally, this meant that in social interactions, participants felt they had to hide their ECT.

Once I’ve told them I’ve had ECT, there are only two reactions I get. […] which is “oh my god, they don’t still do that do they?” […] and the other one is kind of stunned silence and a slow retreat away. (Paula)

Disclosing ECT often left participants feeling othered or inferior: “I felt like I was viewed as less, less of an equal. […] you know the power never evened up again” (Paula).

Difficulties connecting with others and socializing were exacerbated by the forgetting of memories of shared experiences or being unable to build upon conversations: “I was so anxious about meeting up with people […] I knew that I couldn’t remember what we had talked about previously” (Stella). For some participants, their relationships with friends and family were affected as others did not understand or were hurt by the loss of shared memories: “The school friends it’s really difficult […] I can’t remember any of it, and I think they feel kind of hurt” (Flora). This resulted in participants withdrawing socially or masking their difficulties: “I have to like to put a show on all the time, it’s exhausting” (Rose).

##### “It All Just Went Clunk” (Harriet)

Whilst some participants returned from hospital understanding that their memory loss and cognitive changes were due to ECT, others did not make this link, causing confusion: “Just incredibly confusing, I haven’t been able to make any sense of it, it’s it just made no sense” (Harriet). The absence of specific follow-up care meant that participants were not supported to understand the adverse effects of ECT, leaving participants feeling isolated and helpless: “No one does the six-week check-up or the six year or the sixty year […] and for me that’s shocking” (Paula).

Where participants did manage to access appointments, they often felt that professionals dismissed their experiences. Therefore, the emotional impacts of ECT were rarely discussed, leaving participants feeling silenced: “They just didn’t listen!” (Charlotte). Professionals often attributed post-ECT difficulties to the effects of taking psychiatric medication, experiences of depression, or other diagnostic explanations. However, participants did not feel that these explanations were valid reflections of their experiences: “That’s the psychiatrist switch, from saying it’s depression to it was PTSD and that was his blanket explanation for everything” (Harriet).

For Stella and Harriet, professionals saw memory loss as a separate problem to mental health, meaning that they received little support from psychiatric care. They felt abandoned and betrayed: “I don’t really now fit completely into that box if you like, in psychiatry but I also can’t fit into anywhere else. […] you’re just kind of left you know” (Stella). All of which contributed to participants generally avoiding involvement with mental health services, especially psychiatrists.

By chance, three participants, Harriet, Paula, and Dean, heard a radio program discussing adverse experiences of ECT which helped them to comprehend their new reality.

This mind-blowing radio programme […] describing so, such a similar experience to mine and it all, it all just went clunk, and and then I understood why I couldn’t, why I couldn’t read, and I understood that that reading is a memory problem, and that memory is an ECT problem. (Harriet)

##### “It’s Brain Damaged Us” (Rose)

In seeking to understand post-ECT changes, most participants arrived at the meaning that ECT had been traumatizing and had caused brain damage. Furthermore, in considering their brain to have been damaged, participants worried about how future age-related brain degeneration would affect them: “I am closer on the scale to mild cognitive impairment […] than I am to being a typical person and that worries me because I know about neurodegeneration with age” (Charlotte). Three participants had accessed brain scans or neuropsychological tests, which provided evidence that their brains and capacities had measurably changed. These participants were able to discuss cognitive changes and memory loss with more certainty than others.

I have a diagnosis of traumatic brain injury, acquired brain injury in my medical notes, and you know, scans that prove that it’s, that it’s there and a report that says that the injury is consistent with a history of ECT. (Harriet)

##### “My Life Is One of Post-It Notes” (Charlotte)

All participants had significantly adapted their lives to cope with their adverse experiences. The different ways that participants adapted and coped are captured across the sub-themes: New Ways of Being, Prompts and Strategies, and Sharing Experiences.

###### New Ways of Being

Some participants became more dependent on their family and friends to function, and with this increased dependence came an increased sense of vulnerability. Additionally, other participants had to change their way of being in the world; for Stella, this meant connecting with a deeper spirituality to cope with the impacts of ECT: “It’s like certain things feel like they’ve been stripped from me, so I’ve had to try to connect with something deeper” (Stella). Due to difficulties recalling and retaining memories, some participants found that they had to live more in the present moment due to being unable to reminisce or be future-oriented. However, for Flora this meant living more in the past: “I tend to have more memories from before 2008 than I do after. […] that’s why I live in the past.”

###### Prompts and Strategies

All participants experiencing cognitive and memory difficulties had developed ways to navigate through the world with their new disabilities. Some participants were able to use visual or written prompts. However, the context of prompts was important as without enough explanation or detail, they were not helpful to participants later: “My life is one of Post-it notes […] I stick Post-it notes all over places to remind myself of things” (Charlotte). Some participants also found that repetition and/or routines created helpful ways for them to remember more successfully: “I rely quite heavily on procedure and routine. I find that I’m less likely to forget things when they become like non-verbal habits” (Charlotte). Participants also spoke about learning to adapt to daily life through accepting what had happened to them in order to reduce their distress: “It’s happened I can’t change it, and I just have to try and keep going with what I’ve got” (Flora).

###### Sharing Experiences

All participants recognized the benefits of sharing their experiences with others. Some participants had been able to find support from others, sometimes specific to ECT and sometimes through more generic mental health support groups. Sharing stories was mutually beneficial, creating a sense of understanding, connection, and purpose for participants: “Publishing about it and you know agreeing to sort of things like this and talking to people […] is part of the sort of the therapy and sort of coming to terms with what happened” (Dean). However, due to the isolating nature of cognitive and memory difficulties, finding others with similar experiences was hard for some participants: “Even one person, if there was one person nearby who had something similar, that would really help” (Harriet).

### Part 3: How Participants Told Their Story: Structural and Performative Elements

The stories participants told were constructed both from their own memories (depending on the extent of these) and other sources of information, such as personal notes, family members, friends, medical professionals, and medical accounts.

#### Structural Elements

Two parts of Labov and Waletzky’s (1967) five-part structural analysis, abstract (a summary of the events) and complicating action (the actual events of the narrative), were generally present across narratives. However, the other three parts, orientation (information about the setting of the story), evaluation (the reason the story was told), and resolution (the conclusion), were lacking. Narratives were generally sparse in orienting details, contained frequent gaps, missed evaluation of events, and moved between substories without resolution.

Narrative coherence was explored by utilizing Baerger and McAdams (1999) framework. Narratives typically lacked coherence across all criteria. Most narratives became disoriented, with participants losing track of what they were talking about due to not remembering what they had already covered or what they were trying to convey. Three participants required considerable support to structure their stories temporally. As participants recounted their experiences of ECT, there was a noticeable absence of affect; the emotional significance of events was generally elicited through specific prompting later in the narrative interview. Furthermore, across all accounts, the integration of stories of ECT within the larger life story was lacking, especially the integration of ECT with life prior to it. Participants’ stories in this study lacked coherence, integration, and resolution. Lower story coherence has been linked to lower psychological well-being (Baerger & McAdams, 1999).

Narrative meaning-making is understood to emerge over time, where the ongoing processing and recall of autobiographical memory is central (Fivush et al., 2017). It is, therefore, unsurprising, due to the autobiographical memory difficulties reported, that narratives in this study lacked meaning-making. As time since ECT has progressed, if memories can be accessed, participants will have had additional opportunities to create narratives and meaning and make sense of events. Some participants were more clearly able to articulate meanings made about ECT than others; this appeared to vary with the extent of memory loss reported. Regardless, discussion of meaning-making was seldom spontaneously considered within narratives and needed specific prompting.

#### Performative Elements

Participants appeared aware of their difficulty constructing a coherent narrative, discussing concerns about their narrative’s believability and credibility. Throughout interviews, all participants frequently read excerpts from, or offered the researcher access to, medical notes. This offered a way for participants to support, reinforce, and verify their narratives.

## Discussion

This research aimed to explore the stories of people who identify experiencing long-term adverse effects from ECT, contributing to the research field by providing insights into the unfolding of adverse experiences of ECT over time.

Powerlessness, lack of control, and conformity have been common themes found within the existing literature exploring ECT experiences (Johnstone, 1999; Knight et al., 2017). Feeling misunderstood and powerless was prominent across narratives within this research. Participants often felt that they had to accept or were not able to challenge professionals, particularly when feeling desperate and hopeless, leading to conformity. Following ECT, most participants had limited engagement with mental health services, and therefore, they had greater control over how they understood their experiences. Some participants spoke about how powerful medical professionals were in their ability to define experiences using biomedical definitions and treatments, such as ECT, and furthermore, how these biomedical ideas did not always fit with the participant’s own sense-making about their distress.

Participants’ experience of long-term adverse effects of ECT impacted their appraisal of the accuracy and sufficiency of consent information. Despite their memory difficulties, most participants still had access to the written information they had been given, making appraisals possible. Pre-treatment information was perceived as vague and incomplete. This was particularly prominent when considering the impacts of memory loss, with participants feeling uninformed about how extensive and fundamental their memory was to their sense of self, identity, and daily functioning. Seniuk (2018) has explored the impact of ECT from a phenomenological perspective, concluding that memory is more than a collection of discrete experiences; memory is inextricably bound up with our past and future sense of self and how we engage with the world. Participants in this research did not take the term “memory loss” to imply a loss of sense of self post-ECT, and therefore, consent information left them unprepared for this experience and its devastating impacts.

This study adds to the literature exploring experiences of ECT, which has found a significant loss of cognitive abilities and memory (Rose et al., 2003; Wells et al., 2021, 2022). However, what this study found, in addition, was that for some participants, memory loss took time to become apparent. Participants spoke about the significance of returning to familiar environments to realize what they could no longer do and what they had forgotten. Recognition of loss of cognitive abilities took repeated exposure to everyday tasks to realize that they were no longer possible. Uncovering the extensive nature of autobiographical memory loss took exposure to a range of what should be familiar people, places, and photographs. Therefore, the exploration of adverse experiences post-ECT needs to allow for the time it takes people to discover changes.

Woven throughout participants’ narratives were stories of participants feeling misunderstood, misinformed, and silenced by the mental healthcare system, understandings that ECT had caused brain damage, and experiences of ECT as traumatizing. These experiences contributed to participants avoiding, fearing, and losing trust in healthcare systems. Collectively, these adverse experiences of ECT constitute physical and psychological iatrogenic harm. Psychological iatrogenic harm from procedures being experienced as traumatic, including ECT, is beginning to be discussed (Johanssen-Everyday, 2023). However, in addition, within this study, participants’ interactions with mental health professionals, especially the denial or dismissal of adverse experiences post-ECT and absence of follow-up care, were considerably distressing and could constitute further psychological iatrogenic harm. Indeed, avoidance of mental healthcare systems, as discussed by participants in this study, has been found to be a common response to psychiatric iatrogenic harm (Aves, 2024).

An unexpected finding of this research was the extensive impact of loss on participants. Perhaps due to the focus of this research on long-term impacts, meaning-making, and coping, the ongoing nature of loss and far-reaching effects were explored in greater depth. The loss that participants experienced appears to fit with the concept of ambiguous loss (Boss, 1999). Ambiguous loss is defined as a situation of unclear loss that remains unverified and thus without resolution (Boss, 1999). It is often considered in the context of brain injury, where profound changes to a person’s cognition and abilities can result in a scenario where the person is physically present but psychologically altered (Kean, 2010). Additionally, the iatrogenic nature of ECT brain damage may add additional complexity to participants’ experience of loss.

This study highlights important elements of participant experience of ECT consistent with the framework proposed by Wells et al. (2022). This study adds to the framework proposed by Wells et al. (2022) by highlighting the importance of time in the development of people’s experiences of ECT and adding more depth and richness to the emotional and psychological impacts that ECT has on people’s lives. What may be unusual in this study is that some participants in this study had accessed neuropsychological tests and brain scans. Access to these tests appeared to help participants to confirm and accept the presence and permanence of their cognitive difficulties. In the United Kingdom, the provision of follow-up care, especially neuropsychological testing following ECT, is limited, which may explain why this finding has largely been absent from previous research.

Participants struggled to tell coherent stories, often missing orienting details, resolution, and evaluation of events, which was consistent with post-ECT storytelling difficulties recognized by van Daalen-Smith (2011). During interviews, the use of timeline and specific prompts supported participants to tell and explore their story. Vankaen et al. (2021) found that when stories are less coherent, people hearing them are less willing to interact, hold fewer positive feelings, and feel less empathy and trust toward the storyteller. Following ECT, the impacts on participants’ ability to tell a coherent story may contribute to participants not being listened to. It is important that participants’ difficulties in telling stories are recognized, understood, and appropriately supported by clinicians.

### Strengths, Limitations, and Recommendations for Future Research

This study provided an in-depth, rich, and clinically applicable understanding of the long-term adverse experiences of ECT. The use of narrative methodology is a key strength of this research, enabling and supporting the exploration of participants’ experiences with ECT over time. Additionally, the findings of this research have highlighted the need for more longitudinal research that considers the long-term and adverse impacts of ECT. Enquiry is needed into the specific areas of cognitive and memory loss and how these difficulties can impact sense of self and identity. Further research should explore how to support people to understand and cope with adverse experiences of ECT, including neuropsychological testing and considering the iatrogenic nature of harm from ECT.

The sample was homogenous across reasons for and circumstances leading to ECT, ethnicity, and education level. Participants in this study had a high level of education and intellect. On the one hand, this can be considered a strength, as despite the aforementioned storytelling difficulties, participants retained a level of skillful articulation from which to explain their experiences. However, this may mean that the narratives described may not be representative of many people receiving ECT. Future research is needed into the experiences of a much more diverse sample, especially the experiences of minoritized people.

The strategy of recruiting through ECT researchers, who shared the research flyer with online mental health and ECT support groups, can be considered a limitation. Although no participant identified as being involved with a campaign group or being an activist, the exposure to others’ experiences of ECT may have influenced participants’ appraisals of their own experiences. Furthermore, only participants with enough ability and financial resources to access online groups using technology were recruited. Therefore, those experiencing greater adverse effects of ECT or those living in poverty may have been inadvertently excluded.

Due to the focus on post-ECT experiences, this study did not explore participants’ full lifeline in depth. There is a high likelihood that those accessing mental health services will have experienced trauma (Kessler et al., 2010). Therefore, there may be additional impacts of prior life and childhood experiences, particularly traumatic, on the psychological and emotional effects of ECT not discussed in this study that require further exploration.

### Clinical Implications

Findings from this study suggest that ECT can be a traumatic, psychologically and emotionally distressing experience with profound impacts on a person’s sense of self. Participants suggested that diagnoses and psychiatric drug treatment were not always helpful. It is important that people feel empowered to choose what is right for them within their care. It is essential that services offering ECT as a treatment ensure that people are provided with accurate consent information that outlines the true possibility of devastating and permanent side effects. Furthermore, services need to provide environments where participants feel free to consent rather than conform. There is undoubtedly a role for clinical psychologists in psychological formulation before ECT, ensuring the consent process is fully informed and offering neuropsychological testing and psychological support for post-ECT difficulties.

Findings suggest that service development is urgently needed to support people experiencing the adverse effects of ECT, particularly concerning specific long-lasting follow-up care. Negative experiences of ECT need to be recognized as a significant reality for some. Follow-up care requires those involved in the practice of ECT and professionals working in the wider mental health systems to be able to hear and accept a diversity of accounts regarding ECT. Therefore, it is fundamentally important that services create systems where people are safe, supported, and empowered to tell their stories of adverse experiences of ECT.
